# Transstadial Transmission of *Francisella tularensis holarctica* in Mosquitoes, Sweden

**DOI:** 10.3201/eid1705.100426

**Published:** 2011-05

**Authors:** Jan O. Lundström, Ann-Christin Andersson, Stina Bäckman, Martina L. Schäfer, Mats Forsman, Johanna Thelaus

**Affiliations:** Authors affiliations: Uppsala University, Uppsala, Sweden (J.O. Lundström, M.L. Schäfer);; Swedish Defence Research Agency, Umeå, Sweden (A.-C. Andersson, S. Bäckman, M. Forsman. J. Thelaus)

**Keywords:** Mosquitoes, vector-borne infections, culicidae, Francisella tularensis holarctica, bacteria, Aedes sticticus, transstadial transmission, tularemia, Sweden, research

## Abstract

In Sweden, human cases of tularemia caused by *Francisella tularensis* *holarctica* are assumed to be transmitted by mosquitoes, but how mosquito vectors acquire and transmit the bacterium is not clear. To determine how transmission of this bacterium occurs, mosquito larvae were collected in an area where tularemia is endemic, brought to the laboratory, and reared to adults in their original pond water. Screening of adult mosquitoes by real-time PCR demonstrated *F. tularensis* *lpnA* sequences in 14 of the 48 mosquito pools tested; *lpnA* sequences were demonstrated in 6 of 9 identified mosquito species. Further analysis confirmed the presence of *F. tularensis* *holarctica*–specific 30-bp deletion region sequences (FtM19inDel) in water from breeding containers and in 3 mosquito species (*Aedes sticticus*, *Ae. vexans*, and *Ae. punctor*) known to take blood from humans. Our results suggest that the mosquitoes that transmit *F. tularensis* *holarctica* during tularemia outbreaks acquire the bacterium already as larvae.

Outbreaks of tularemia are caused by the bacterium *Francisella tularensis* *holarctica* throughout the Northern Hemisphere and by *F. tularensis* *tularensis* in North America only. Routes of infection include transmission from blood-sucking arthropods and through contact with infected dead or live animals, as well as from aerosols, dust, and water (*1*). Two primary disease manifestations, ulceroglandular and glandular tularemia, are associated with vector-borne transmission of the bacterium (*1*). Traditionally, mosquitoes are considered the primary vectors of *F. tularensis* *holarctica* to humans in Russia and Scandinavia (*2**–**4**,**5*). Moreover, mosquito-borne transmission of tularemia may be becoming more common in central Europe; evidence shows that this infection has reemerged during the past decade (*6**,**7*).

The ulceroglandular form of tularemia is by far the most common in Sweden; most human cases occur in late summer and early fall and are assumed to be transmitted by mosquitoes (*4**,**8*). A total of 5,754 human cases of tularemia were reported during 1931–1993, and the incidence of infection varies greatly among these years, ranging from a few cases in some years to >2,700 cases during 1967 (*8*). In the Örebro area of central Sweden, widespread mosquito-associated tularemia outbreaks first occurred during 2000 and 2003 (*4**,**5*), after which human cases have continued to occur in this new area where tularemia is endemic (www.smi.se/statistik/harpest). However, how vector mosquitoes acquire the bacterium is still not clear.

The demonstrated ability of *F. tularensis holarctica* strains to survive in association with protozoa indicates that ubiquitous aquatic protozoa might be an important environmental reservoir for the bacterium (*9**–**11*). Moreover, mosquito larvae, mainly the species *A. sticticus* and other floodwater mosquitoes, exert a predatory effect on aquatic protozoan populations (*12*). These factors indicate that mosquito larvae may be exposed to *F. tularensis* in their natural aquatic environment. We investigated the natural occurrence of *F. tularensis* in mosquitoes hatched from larvae collected in an area where tularemia was endemic. Because of unknown mechanisms, the bacterium *F. tularensis* is extremely difficult to isolate directly from environmental samples. Thus, our study focuses entirely on molecular techniques.

## Materials and Methods

### Sample Collection

Mosquito larvae were sampled on August 28, 2008, in Örebro, an area where tularemia is endemic. Nine human tularemia cases (3.24/100,000 persons) were reported from Örebro County in June–September 2008 (www.smi.se/statistik/harpest). Two sampling locations were selected, Ormesta (WGS84; 59°16′12′′N, 15°16′48′′E) and Vattenparken (WGS84; 59°16′55′′N, 15°15′00′′E), on the basis of a geographic distribution study of human tularemia cases in the area (*13*). Both locations are situated at Lake Hjälmaren near the city of Örebro and are characterized by lush vegetation of reed belts and deciduous trees and bushes. Using a standard dipper, we collected mosquito larvae from shallow temporary water bodies in the transition zone between reed and willow bush habitats (Ormesta 1), in the deciduous forest (Ormesta 2), and in a ditch covered by bush and grass (Vattenparken).

Mosquito larvae from each water body were reared to adults in their original pond water (Ormesta 1, containers A and B; Ormesta 2, containers C, D, E, F, and G; and Vattenparken, container H). At the start of this study, time-zero samples of the original water from each container were collected and stored at –20°C. Emerging adult mosquitoes were collected by a mechanical aspirator, killed by freezing, and stored at –80ºC until species was identified. During identification, adult mosquitoes were kept cold on a chill table, illuminated by a cold light lamp, and identified to species based on morphologic features. Identified mosquitoes were sorted by area, species, and sex in pools of 1–50 specimens and returned to the –80ºC freezer.

### DNA Extraction from Mosquitoes and Water Samples

For DNA extraction, 10 μL of 2.8 M NH_4_OH solution and 450 mg each of 1.0-mm and 0.1-mm silica beads were added to each pooled mosquito sample. Samples were homogenized for 60 s (BeadBeater FastPrep; BioSpec Products, Inc., Bartlesville, OK, USA). The homogenized samples were incubated at room temperature for 15 min, and 60 μL of sterile water was added. DNA extraction was performed by using SoilMaster DNA Extraction Kit (Epicentre Biotechnologies, Madison, WI, USA) according to the manufacturer’s instructions. The resulting DNA pellet was resuspended in 60 μL Tris EDTA buffer.

DNA extraction from water samples was performed as previously described (*14*). Two milliliters of each water sample was centrifuged at 16,000 × *g* for 1 h, 1.9 mL of the resulting supernatant was discarded, and DNA was extracted from the remaining volume by using a SoilMaster DNA Extraction Kit (Epicentre Biotechnologies).

### PCR Analysis of Mosquitoes and Water

Mosquitoes and water samples were screened for *F. tularensis* by using a modified real-time PCR SYBR-based assay (Quanta BioSciences, Gaithersburg, MD, USA) for detection of the *F. tularensis*-specific *lpnA* gene. The PCR assay was modified from the methods of Thelaus et al. (*11*). Each reaction consisted of 1 µL template, 1× Quanta PerfeCTa SYBR Green FastMix (Quanta BioSciences), 400 nmol/L for each of the *lpnA*2F/R-primers (5′-CGCAGGTTTAGCGAGCTGTT-3′ and 5′-GAGCAGCAGCAGTATCTTTAGC-3′), and Milli-Q (Millipore, Billerica, MA, USA) up to 20 µL. An initial denaturation at 95°C for 5 min was followed by 40 cycles at 95°C for 10 s, 60°C for 30 s, and a melt curve 60°C–95°C on Mastercycler (Eppendorf, Hamburg, Germany).

Mosquitoes and water samples then underwent a *F. tularensis* *holarctica*–specific PCR, based on the 30-bp–deletion region FtM19 and using the FtM19InDelF/R primer pair, and modified from PCR (*14*). Each reaction consisted of 1–3 µL templates, 1x SsoFast EvaGreen Supermix (Bio-Rad, Hercules, CA, USA) 400 nmol/L for each of the primers FtM19Indel F/R (5′-GAATTACATAAAGTTCATGGTCCAGTAC-3′ and 5′-GTTTCAGAATTCATTTTTGTCCGTAA-3′) and Milli-Q (Millipore) water to give a final volume of 20 µL. An initial denaturation at 98°C for 2 min was followed by 50 cycles at 98°C for 5 s, 60°C for 5 s, and a melt curve 65°C–95°C on a Bio-Rad CFX96. Positive control mixtures, using DNA from *F. tularensis holarctica* and negative control mixtures without a template, were included in each PCR run.

### Sequencing

The *lpnA* gene and FtM19InDel amplicons were cloned with TOPO TA cloning kit PCR4 (Invitrogen, Carlsbad, CA, USA) and sequenced. The acquired sequences were deposited in GenBank under accession nos. GY97987–GU97997 and HQ289871–HQ289876 (*lpn*A and FtM19InDel, respectively).

## Results

### *F. tularensis* in Mosquitoes Hatched from Field-collected Larvae

**Figure 1:**
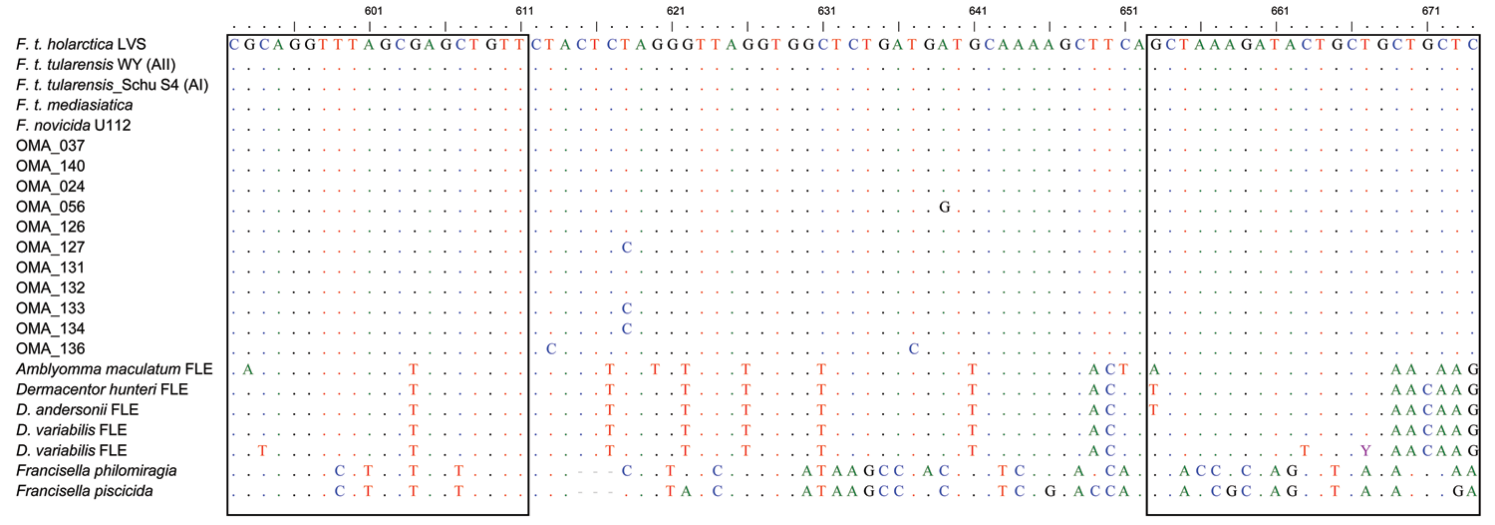
Multiple alignment of the 11 *Francisella* *lpnA* sequences obtained from mosquitoes in Sweden (hatched from field-collected larvae) with previously published sequences of *Francisella* species and subspecies, and *Francisella*-like endosymbionts (FLE). Boxed nucleotides represent target sequences of *lpnA* primers. The nucleotide positions 592–674 refer to *F. tularensis holarctica* live vaccine strain (LVS). Colors indicate individual nucleotides to clearly delineate those diverging from the *F. tularensis holarctica* LVS sequence. Reference sequences from GenBank; *F. tularensis holarctica* LVS (M32059), *F. tularensis tularensis* strain WY96-3418 (CP000608), *F. tularensis tularensis* strain Schu S4 (NC_006570), *F. tularensis mediasiatica* strain FSC147 (NC_010677), *F. tularensis novicida* strain U112 (CP000439), *Amblyomma maculatum* FLE (AY375422), *Dermacentor hunteri* FLE (AY375417), *D. andersonii* FLE (AY375413), *D. variabilis* FLE (AY375420), *D. variabilis* FLE (AY375421), *F. philomiragia* (AY243030), and *F. piscicida* (DQ825765).

The 334 adult mosquitoes of 9 species hatched from mosquito larvae collected in the tularemia-endemic Örebro area were analyzed in 48 pools; 14 pools (29%) were positive for the *F. tularensis* *lpnA* gene (Table 1). Eleven of the 14 *lpnA*-positive samples were possible to sequence (Figure 1). All obtained sequences showed high sequence similarity (>97%) with *F. tularensis* in alignment with published sequences from representatives of subspecies of *F. tularensis* and their closest known relatives (i.e., *Francisella*-like endosymbionts).

**Table 1:** Presence of *Francisella tularensis* *lpnA* and *F. tularensis holarctica*–specific 30-bp–deletion region (FtM19InDel) in female and male mosquitoes, Örebro area, Sweden*

Group	Species	Female mosquitoes				Male mosquitoes		
		Pools	*F. tularensis*	*F*. *tularensis holarctica*		Pools	*F. tularensis*	*F. tularensis holarctica*
2a	*Aedes communis*	5 (*10*)	1	ND		–	–	–
2a	*Ae. intrudens*	2 (*2*)	ND	ND		–	–	–
2a	*Ae. punctor*	5 (*5*)	1	1		–	–	–
2a	*Aedes* spp.†	–	–	–		10 (31)	2	ND
2b	*Ae. cinereus*	7 (135)	3	ND		7 (109)	2	ND
2b	*Ae. sticticus*	4 (*17*)	2	ND		2 (*12*)	1	1
2b	*Ae. vexans*	2 (2)	1	1		–	–	–
1c	*Culiseta alaskaensis*	1 (*1*)	ND	ND		–	–	–
1c	*Cs. annulata*	1 (*1*)	ND	ND		–	–	–
1d	*Culex pipiens/torrentium*	1 (*5*)	ND	ND		1 (*4*)	1	1
Total		28 (178)	8	2		20 (156)	6	2

*Mosquitoes were collected as larvae in the Örebro area, central Sweden, reared to adults, and analyzed by real-time PCR in pools of up to 50 specimens. Numbers in parenthesis refer to total specimens within pools. ND, not detected; –, not analyzed. †Male mosquitoes identified as *Aedes* spp. could belong to any species within functional group 2a and were not identified further. Mosquito functional groups are as defined by Schäfer et al. (*15*).

We observed no difference in the *F. tularensis* detection rate between male (6/20 pools, 30%) and female (8/28 pools, 29%) mosquitoes (Table 1). Using the definitions in Schäfer et al. (*15*), we determined that the mosquito species belonged to 4 of 10 defined mosquito functional groups. Functional group 2a (snow-pool mosquitoes) and functional group 2b (floodwater mosquitoes) constitute most of the mosquitoes tested. Notably, the *F. tularensis* detection rate in female floodwater mosquitoes (6/13 pools, 46%) was higher than in female snow-pool mosquitoes (2/12 pools, 17%).

### *F. tularensis* in Water

Water samples from 5 of the 8 water containers used for rearing were positive for the *F. tularensis lpnA* gene (Table 2). Four of these containers yielded mosquitoes that were positive for the *F. tularensis lpnA* gene. However, 3 water containers used for rearing that were negative for the *F. tularensis lpnA* gene, all yielded adult mosquitoes positive for the *F. tularensis lpnA* gene. Thus, there was no correlation between detection of *F. tularensis* in water from a specific container used for rearing and detection of *F. tularensis* in adult mosquitoes hatched from the container (Table 2).

**Table 2:** Presence of *Francisella tularensis* *lpnA* and *F. tularensis* *holarctica*–specific 30-bp–deletion region in water samples and in pools of mosquitoes hatched from larvae collected in the Orebro area, Sweden*

Pond	Water				Mosquitoes		
Container	*F. tularensis*	*F. tularensis holarctica*	Pools	*F. tularensis*	*F. tularensis holarctica*
Ormesta 1	A	Yes	Yes		2 (*2*)	1	ND
	B	Yes	ND		6 (28)	ND	ND
Ormesta 2	C	ND	ND		6 (74)	2	ND
	D	Yes	ND		9 (79)	4	3
	E	ND	ND		6 (45)	3	ND
	F	Yes	ND		8 (61)	2	ND
	G	ND	ND		5 (27)	1	ND
Vattenpark	H	Yes	Yes		6 (*18*)	1	1
Total	8	5	2		48 (334)	14	4

*Water samples were collected from each mosquito-breeding container on arrival at the laboratory. Numbers in parentheses refer to number of mosquito specimens within pools. Sampling locations are Ormesta and Vattenpark. ND, not detected.

### Detection of *Francisella tularenis* spp. *holarctica*

**Figure 2:**
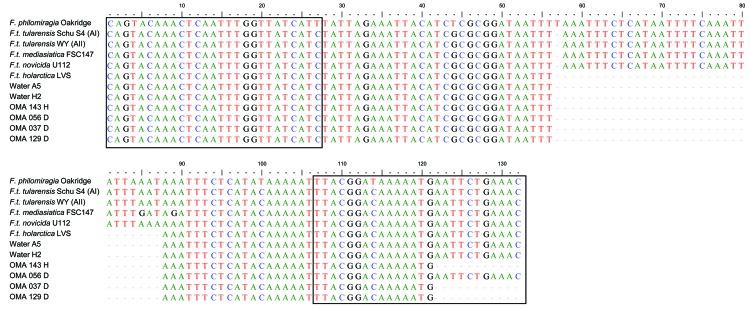
Multiple alignments of the 11 *Francisella* *lpnA* sequences (designated OMA_xxx) obtained from mosquitoes and water samples with previously published sequences of *Francisella* species and subspecies. Boxed nucleotides represent target sequences of FtM19InDel primers. In this alignment the *F. tularensis* subsp. *holarctica* specific deletion is located from position 57 to 87. Colors indicate individual nucleotides. Reference sequences from GenBank: *F. tularensis holarctica* LVS(M32059), *F. tularensis* subsp. *tularensis* strain WY96-3418 (CP000608), *F. tularensis* subsp. *tularensis* strain Schu S4 (NC_006570), *F. tularensis mediasiatica* strain FSC147 (NC_010677), *F. tularensis novicida* strain U112 (CP000439), and *F. philomiragia* (AY243030).

Using the FtM19InDel primers, we detected sequences specific for *F. tularensis* *holarctica* in 2 of the 8 water samples analyzed and 4 of the 48 mosquito pools (Figure 2; Table 2). Mosquito species positive for *F. tularensis holarctica* sequences were *A. punctor* (snow-pool mosquito), *Ae. vexans*, *Ae. sticticus* (both floodwater mosquitoes), and *Culex pipens*/*torrentium* (Table 1).

## Discussion

We detected *F. tularensis* *holarctica* DNA in adult mosquitoes hatched from field-collected larvae sampled in an area in Sweden endemic for tularemia. This finding suggests that mosquitoes came in contact with the causative agent of the disease, *F. tularensis* *holarctica*, in the aquatic habitat of the mosquito larvae. We have previously shown that *F. tularensis holarctica* persists in natural aquatic environments between outbreaks (*14*) and in association with protozoa (*10**,**11*). Mosquito larvae of floodwater mosquitoes (i.e., *A. sticticus*) are predators on protozoa in temporary wetland environments (*12*). Our results suggest natural transstadial transmission of *F. tularensis* *holarctica* from its water reservoir via female mosquitoes to their vertebrate blood-meal hosts, including humans. The observation of water containers negative for *F. tularensis* that yielded mosquitoes positive for the bacterium and vice versa suggests that mosquitoes were truly positive for *F. tularensis* and not cross-contaminated with water that tested positive; however, we cannot exclude varying sensitivity of the real-time PCR analysis for water and mosquito samples. Further studies of the tissue tropism of the bacterium within the mosquito body are needed to confirm how *F. tularensis* *holarctica* is transmitted by mosquito vector.

Transstadial transmission by mosquitoes after ingestion of pathogenic microorganisms as larvae has previously been shown for Rift Valley fever virus (*16*). The virus was transstadially transmitted to emerging adult mosquitoes (laboratory strain of *Cx. pipiens* and natural strains of *Ae. circumluteolus* and *Abydosaurus mcintoshi* from Kenya) that were capable of transmitting Rift Valley fever virus to hamsters (*16*). Transstadial transmission of *F. tularensis* subspecies has also been reported in several species of ticks (*3**,**17*).

In a recent study, transmission of *F. tularensis* *novicida* was tested in laboratory strains of the tropical mosquitoes *Anopheles gambiae* and *Ae. aegypti* (*18*). However, the bacterium was not transmitted transstadially to adult mosquitoes, and female mosquitoes exposed to *F. tularensis* *novicida* in a blood meal were not able to transmit the bacterium to mice. Results of this study, along with our results, contribute to the growing body of data that indicate differences in the ecology, including vectors and reservoirs, of *Francisella* species, subspecies, and even populations (*3**,**14**,**19**,**20*).

We detected *F. tularensis* DNA in 29% of the pooled samples of adult mosquitoes hatched from field-collected larvae, indicating that transmission of the bacterium from water can generate a relatively high proportion of infected adult mosquitoes in an area endemic for tularemia. In line with our results, the *F. tularensis* *fopA* gene was detected in 30% of mosquito pools sampled in Alaska (*18*). However, further studies of host-seeking female mosquitoes in areas where tularemia is endemic are required to identify the range of mosquito species naturally infected with *F. tularensis* *holarctica* and the temporal distribution of the bacterium in these potential vector species in relation to the onset of outbreaks.

We detected *F. tularensis holarctica* in the floodwater mosquito species *Ae. sticticus* and *Ae. vexans,* the snow-pool mosquito species *Ae. punctor*, and a mixture of *Cx. pipiens* and *Cx. torrentium* mosquitoes. The 3 *Aedes* spp. mosquitoes feed primarily on mammals and commonly take blood meals from humans; the 2 *Culex* spp. mosquitoes feed on birds (*15*). With respect to blood-feeding habits, and the detection of *F. tularensis holarctica* DNA, all 3 *Aedes* spp. mosquitoes are potential vectors for transmission of *F. tularensis holarctica* to humans. Human cases of tularemia in Sweden (ulceroglandular and glandular) occur mainly in late summer and fall (*13*), a period when floodwater mosquito species are dominating the Swedish mosquito fauna (*21*). Notably, the detection rate of *F. tularensis* was higher in floodwater pools of female mosquitoes (46%) than in snow-pool pools of female mosquitoes (17%). The observation that *F. tularensis* *holarctica* occur in the floodwater mosquito *Ae. sticticus* is especially noteworthy because this nuisance species is now increasing its geographic range within Sweden (*22*).

We suggest that the transmission of the bacterium *F. tularenis holarctica* via blood-feeding mosquitoes to humans in areas of Sweden where tularemia is endemic originates from the aquatic habitat of mosquito larvae. However, further studies are needed to confirm transmission of the bacterium from its aquatic reservoir by blood-feeding female mosquitoes to their vertebrate hosts. The finding of *F. tularensis* *holarctica* DNA in adult mosquitoes, hatched from larvae collected in an area where tularemia is endemic, indicates that disease transmission in outbreaks originates in the pond habitats of the mosquito larvae.
